# Treasure of the Past VII: Measurement of the Thickness and Refractive Index of Very Thin Films and the Optical Properties of Surfaces by Ellipsometry^1^

**DOI:** 10.6028/jres.106.025

**Published:** 2001-06-01

**Authors:** Frank L. McCrackin, Elio Passaglia, Robert R. Stromberg, Harold L. Steinberg

## Abstract

The use of the ellipsometer for the measurement of the thickness and refractive index of very thin films is reviewed. The Poincaré sphere representation of the state of polarization of light is developed and used to describe the reflection process. Details of the operation of the ellipsometer are examined critically. A computational method is presented by which the thickness of a film of known refractive index on a reflecting substrate of known optical constants may be calculated directly from the ellipsometer readings. A method for computing both the refractive index and thickness of an unknown film is also developed. These methods have been applied to the determination of the thickness of an adsorbed water layer on chromium ferrotype plates and on gold surfaces. In the former case the thickness was 23 to 27 Å, and in the latter was 2 to 5 Å. The measurement of the thickness and refractive index of barium fluoride films evaporated on chromium ferrotype surfaces is used as an illustration of the simultaneous determination of these two quantities.

## 1. Introduction

Ellipsometry is a convenient and accurate technique for the measurement of thicknesses and refractive indexes of very thin films on solid surfaces and for the measurement of optical constants of reflecting surfaces. The lower limit of film thicknesses that can be studied by ellipsometry is at least an order of magnitude smaller than can be studied by other means such as interferometry. Artifacts such as those caused by vacuum in the case of electron microscopy are not encountered. Neither interferometry nor electron microscopy are adaptable, as is ellipsometry, to the study of films under liquids, and only ellipsometry will give the index of refraction of films of unknown thickness.

The technique of ellipsometry is concerned with the measurement of changes in the state of polarization of light upon reflection from a surface. For a clean reflecting surface the optical constants of the surface and the reflection coefficients of the system may be calculated from these changes. A thin transparent film on the reflecting surface causes additional changes from which the thickness and refractive index of the film may be determined.

The principle of the ellipsometer has been described by several authors [e.g., 1–7]^2^ and a bibliography of the theoretical contributions of many authors, starting with the original equations of Drude [1] is given by Winterbottom [2]. This paper will describe certain measurement techniques and an application of the exact solution of the algebraic equations of Drude to the measurement of optical constants of surfaces and thickness and refractive index of thin films covering the surfaces. A generally applicable computational method is described. The complex and lengthy calculations required to solve the equations involved have been programmed for an electronic computer. Measurements carried out on chrome and gold surfaces with and without films will also be described.

In addition, the Poincaré sphere representation of the state of polarization of light will be developed since this is the most useful representation for the consideration of the effects occurring on reflection.

## 2. Representations of Elliptically Polarized Light

**Figure 1:**
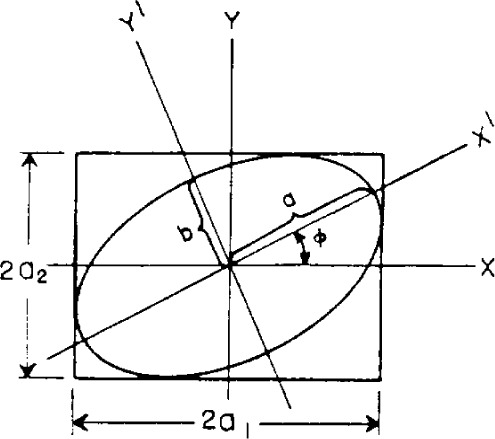
The locus of the electric vector for elliptically polarized light in a plane normal to the direction of propagation. The coordinate axes *XY* and *X′Y′* are both convenient for the description of the ellipse.

The state of polarization of elliptically polarized light may be described in many ways. For this purpose, consider a light wave traveling along the *Z* axis of a coordinate system. The electric vector of the wave is then given by:

$$\begin{array}{l} E_{X}=a_{1}\;\cos\left(\tau +\delta_{1}\right)\\ E_{Y}=a_{2}\;\cos\left(\tau +\delta_{2}\right) \end{array}$$ (1)

where $\tau =\omega \left(t-\frac{z}{\nu }\right)$, *ω* and *ν* are the angular frequency and linear velocity of the light, respectively, and the total amplitude is the vector sum of *a*_1_ and *a*_2_. The polarization can be described by the amplitudes, *a*_1_ and *a*_2_, and the phase difference, *δ*=*δ*_2_−*δ*_1_, of the components. As is well known, in a stationary plane whose normal is parallel to the *Z* axis the locus of the end of the electric vector is the ellipse shown in figure 1.

The ellipse, however, may also be described in relation to coordinates *X′* and *Y′* along the axes of the ellipse. Thus, the inclination *φ* of these coordinates and the semiaxes *a* and *b* of the ellipse also describe the polarization of the light.

It will be convenient to introduce auxiliary angles *α* and χ defined by

$$\tan\;\alpha =\frac{\alpha_{2}}{\alpha_{1}}$$ (2)

$$\tan\;\chi =\pm \frac{b}{a}.$$ (3)

The numerical value of tan χ represents the ratio of the minor to major axes of the ellipse and the sign of χ distinguishes the two senses in which the ellipse may be described. The angles χ and *φ* are called the ellipticity and azimuth of the light, respectively, and the representation may be made either in terms of *a*_1_ *a_2_*, and *δ; α, δ*, and the total amplitude; or *φ*, χ, and the total amplitude.

Another representation of the state of polarization, and one which leads quite naturally to the Poincaré sphere is by parameters which all have the same physical dimensions, called Stokes parameters [8]:

$$\begin{array}{l} S_{0}=a_{1}^{2}+a_{2}^{2}\\ S_{1}=a_{1}^{2}-a_{2}^{2}\\ S_{2}=2a_{1}a_{2}\;\cos\;\delta \\ S_{3}=2a_{1}a_{2}\;\sin\;\delta . \end{array}$$ (4)

The parameter *S*_0_ is proportional to the intensity of the wave and is related to the other parameters by the identity $S_{0}^{2}=S_{1}^{2}+S_{2}^{2}+S_{3}^{2}$, so that the Stokes parameters are not all independent.

It has been shown [9,10] that the Stokes parameters are also given by

$$\begin{array}{l} S_{1}=S_{0}\cos2\chi \cos2\varphi \\ S_{2}=S_{0}\cos2\chi \sin2\varphi \\ S_{3}=S_{0}\sin2\chi . \end{array}$$ (5)

**Figure 2:**
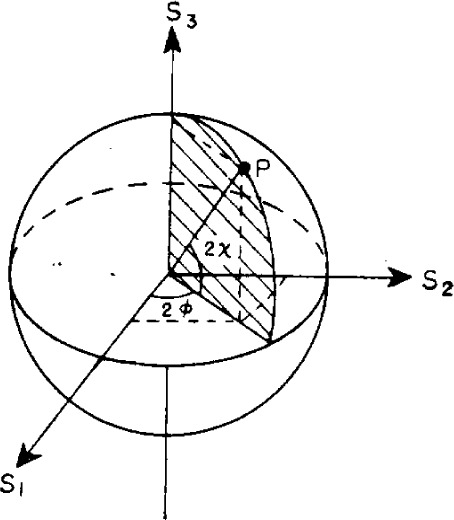
Representation of polarized light onthe Poincaré sphere. The point *P* represents light with ellipticity χ and azimuth *ψ*. The cartesian coordinates are Stokes parameters (after Born and Wolf [9]).

Therefore, the state of polarization may be represented by a point on a sphere of radius *S*_0_ by the spherical coordinates *S*_0_, 2*φ*, 2*χ*. This sphere is called the Poincaré sphere. Alternatively, the Stokes parameters *S*_1_ *S*_2_, and *S*_3_ may be used as Cartesian coordinates to describe the polarization. This representation is illustrated in figure 2.

Since the intensity of the light is of secondary importance for use of the ellipsometer, the projection of the point on a sphere of unit radius is convenient. The coordinates are then given by

$$\begin{array}{l} s_{0}=1\\ s_{1}=\frac{1-\tan^{2}\alpha }{1+\tan^{2}\alpha }=\cos2\chi \cos2\varphi \\ s_{2}=\frac{2\tan\alpha \cos\delta }{1+\tan^{2}\alpha }=\cos2\chi \sin2\varphi \\ s_{3}=\frac{2\tan\alpha \sin\delta }{1+\tan^{2}\alpha }=\sin2\chi . \end{array}$$ (6)

Light of a given state of polarization is therefore represented by a point on the sphere, with the polar angles 2 *φ* and 2 χ representing, respectively, twice the azimuth and twice the ellipticity of the light. For example, for plane polarized light, the ellipticity χ is zero, and *s*_3_ = 0. Therefore, plane polarized light is represented by points on the equator of the Poincaré sphere. Also, the ellipticity of circularly polarized light is 45°, whence *s*_1_=*s*_2_=0, 2χ=±*π*/2; thus, circularly polarized light is represented by the poles of the sphere.

The Poincaré sphere may be used to represent elliptically polarized light with respect to any physical axes, say *X″* and *Y″* at an angle *φ″* with the *XY* axis. The ellipticity χ is not dependent on the choice of axes and the azimuth of the light is changed by χ*″*. Thus the *S*_1_ and *S*_2_ axes must be rotated an angle 2 *φ″* around the *S*_3_ axis.

**Figure 3:**
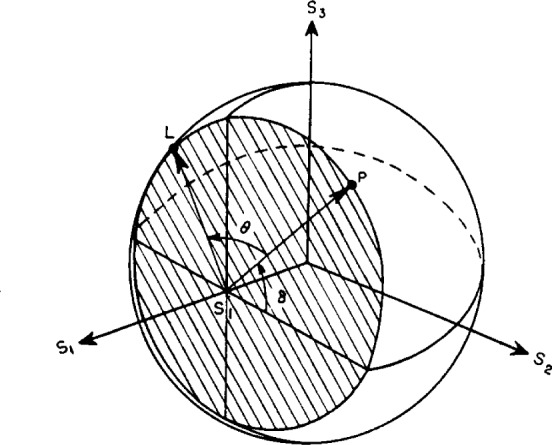
*The intersection of the Poincaré sphere and a plane normal to the* S_1_ *axis at s_1_ = (1−tan^2^α)/(1 + tan^2^α) for the consideration of the effect of a doubly refracting plate with retardation θ on light with ellipticity represented by* P. The plate rotates *P* to *L*. The fast axis of the plate is along *S*_1_.

The Poincaré sphere is convenient for consideration of the effects of doubly refracting plates and reflection on the state of polarization of a light beam. For example, let the polarized wave represented in figure 1 pass through a doubly refracting plate of relative phase retardation *θ*. Choose the *X* and *Y* axes in figure 1 along the slow and fast axes of the plate. Since the azimuth of the fast axis of the plate is zero, it is represented by the positive *S*_1_ axis. In this case there will be no change in the amplitudes *a_1_* and *a_2_* of the components of the light, and therefore no change in *α* or in *s*_1_ as shown by eq (6), the only effect being to change the relative phase of the components by *θ*. Thus, upon passing through the plate the point representing the polarization will remain on the curve representing constant *s*_1_, or on the intersection of the sphere and the plane *s*_1_= constant. The intersection of the Poincaré sphere by a plane perpendicular to the *S*_1_ axis at *s*_1_, shown in figure 3, is a circle of radius $\frac{2\tan\alpha }{1+\tan^{2}\alpha }$ The light incident on the plate is represented by the point *P*, and after passing through the plate is represented by the point *L*. The effect of the doubly refracting plate is seen to turn points on the sphere about the *S*_1_ axis by an angle *θ*. If the plate had been oriented with its fast axis at an azimuth *φ*′ with respect to the *XY* axes, the rotation by *θ* would have been about the line from the center of the sphere and the point 2 *φ*′ on the equator.

Reflection at a metal surface may be represented similarly. For reflection, the incident light wave is resolved into components in the plane of incidence and normal to the plane of incidence (the plane of the surface). The process of reflection introduces a phase difference Δ between these two components, and changes the ratio of their amplitudes by a factor tan *ψ* that is, tan *ψ* is a measure of the relative absorption of the two components. Thus, the ratio of the reflection coefficient for light polarized in the plane of incidence to that for light polarized in the plane of the surface is given (3) by

$$\rho =\frac{r_{p}}{r_{s}}=\tan\psi e^{j\Delta }$$ (7)

where *ρ* is the ratio of the reflection coefficients *r_p_* and *r_s_*, and *ψ* and Δ are functions of the optical constants of the surface, the wavelength of the light used, the angle of incidence, and, for a film covered surface, the thickness and refractive index of the film. (See also eqs 40 to 46.)

For consideration of the reflection process on the Poincaré sphere, represent the light with respect to Cartesian coordinates with the *X* and *Y* axes in and normal to the plane of incidence and the *Z* axis in the direction of propagation. Then tan *ψ=a_s_/a_p_*. The Stokes parameters of the light before reflection are given by eq (6) and after reflection are

$$\begin{array}{l} s_{1}^{\prime \prime }=\frac{1-\tan^{2}\alpha \cot^{2}\psi }{1+\tan^{2}\alpha \cot^{2}\psi }\\ s_{2}^{\prime \prime }=\frac{2\tan\alpha \cot\psi \cos\left(\delta +\Delta \right)}{1+\tan^{2}\alpha \cot^{2}\psi }\\ s_{3}^{\prime \prime }=\frac{2\tan\alpha \cot\psi \sin\left(\delta +\Delta \right)}{1+\tan^{2}\alpha \cot^{2}\psi } \end{array}$$ (8)

**Figure 4:**
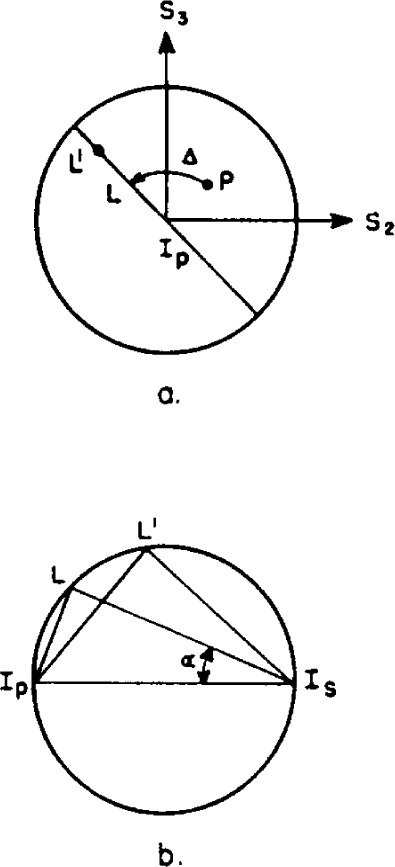
Representation of metallic reflection on the Ponicaré sphere. The azimuth of the plane of incidence is represented by *I_p_* and the plane of the surface by *I*_s_,. In part a the view is along the *S*_1_ axis. The point *P* is rotated to *L* around the *S*_1_ axis by the retardation Δ of the surface. Relative absorption of the components in and normal to the plane of incidence causes translation of *L* to *L*′ along the great circle *I_p_−L*. The view in *b* is normal to this great circle.

The reflection is represented on a Poincaré sphere in figure 4. The point *I_p_* represents the intersection of the axis *S*_1_ with the sphere and has the Cartesian coordinate *s*_1_ = l, *s*_2_=0, *s*_3_=0. By eq (6) this corresponds to zero ellipticity, *χ*, and azimuth, *φ*, so it represents the orientation of the *X* axis, or the plane of incidence. Likewise, the point *I*, has Cartesian coordinates (−1,0,0), so it corresponds to zero ellipticity and an azimuth *π/2*. Therefore, the point *I_s_* represents the orientation of the *Y* axis or plane of the surface. The point *P* representing the polarization of the incident light is rotated around the *S*_1_ axis through an angle Δ, the phase change produced by reflection, to the point *L*. The Stokes parameters of *L* are

$$\begin{array}{l} s_{1}^{\prime }=\frac{1-\tan^{2}\alpha }{1+\tan^{2}\alpha }\\ s_{2}^{\prime }=\frac{2\tan\alpha \cos\left(\delta +\Delta \right)}{1+\tan^{2}\alpha }\\ s_{3}^{\prime }=\frac{2\tan\alpha \sin\left(\delta +\Delta \right)}{1+\tan^{2}\alpha } \end{array}$$ (9)

In addition, the relative absorption of the components translates the point *L* to the point *L*′, the Stokes parameters for which are given by eqs (8).

From eqs (8) and (9) we have $S_{3}^{\prime \prime }/S_{2}^{\prime \prime }=S_{3}^{\prime }/S_{2}^{\prime }=\tan\left(\delta +\Delta \right)$. Points *L* and *L*′ will, therefore, lie on the great circle given by the locus of points on the sphere with s_3_/s_2_=tan (*δ+*Δ). This locus is shown in figures 4a and 4b and is given by the intersection of the sphere with a plane passing through the point *L* and normal to the axis *S*_1_. Thus the point *L* must be moved along the great circle defined above to point *L*′ to represent the polarization of the reflected light. The position of *L*′ on this great circle will now be derived. The Cartesian coordinates of *I_p_* and of *I_s_* are (1,0,0) and (−1,0,0), respectively, and the coordinates of *L* are given by eqs (9). Therefore, the length of the chords *I_p_L* and *I_s_L* are

$$\begin{array}{l} I_{p}L=\frac{2\tan\alpha }{{\left(1+\tan^{2}\alpha \right)}^{1/2}}\\ I_{s}L=\frac{2}{{\left(1+\tan^{2}\alpha \right)}^{1/2}} \end{array}$$ (10)

so

$$\frac{I_{p}L}{I_{s}L}=\tan\alpha .$$ (11)

The angle *I_p_LI_s_* is a right angle since it is inscribed in a semicircle. Therefore, the angle *I_p_I_s_L* is equal to *α*, and it is clear by eq (2) that the tangent of this angle is equal to the ratio of the amplitudes of the components of the incident light.

The position of *L*′ is determined by the angle *I_p_I_s_L*′. The tangent of this angle again gives the ratio of the amplitudes of the components of the reflected light, which is now equal to tan *α*/tan *ψ*. Therefore,

$$\frac{I_{p}L^{\prime }}{I_{s}L^{\prime }}=\tan\left(I_{p}I_{s}L^{\prime }\right)=\tan\alpha /\tan\psi$$ (12)

and the chord *I_p_L*′ may be calculated as

$$I_{p}L^{\prime }=\frac{2}{{\left(1+\cot^{2}\alpha \tan^{2}\psi \right)}^{1/2}}.$$ (13)

Moreover, by eqs (11) and (12), we have

$$\tan\psi =\frac{I_{p}L/I_{s}L}{I_{p}L^{\prime }/I_{s}L^{\prime }}$$ (14)

and, in general, the ratio of the chords between a point representing the state of polarization of a given light wave and any two diametrically opposed points on the sphere is equal to the ratio of the amplitudes of the components when the electric vector of the light is resolved along axes represented by these two opposed points.

The Poincaré sphere representation of the reflection of light from a surface with known complex reflection coefficient tan *ψe^j^*^Δ^ may now be summarized. The point *P*, representing the state of polarization of the incident light is moved by the reflection process through an angle Δ on the sphere in a plane normal to the *S*_1_ axis to the point *L*. The chords *I_p_L* and *I_s_L* are measured and tan *α* calculated by eq (11). Finally the angle *I_p_I_s_L*′ is calculated from eq (12), or the chord *I_p_L*′ is calculated from eq (13), thus determining the state of polarization of the reflected light. We shall show later how this representation may be used conveniently for the calculation of the effects occurring on reflection. First, however, we give a brief description of the instrument.

### 2.1. Instrument

**Figure 5:**
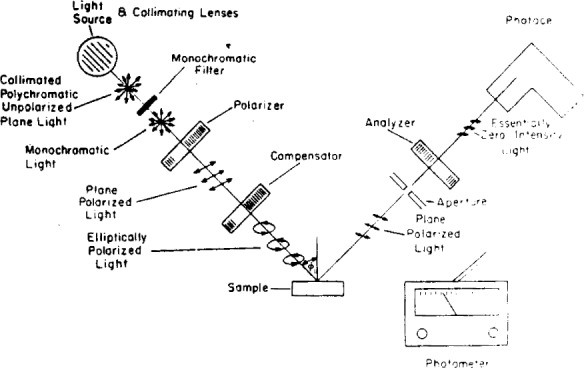
The component parts of an ellipsometer

Figure 5 shows the various components of an ellipsometer. Collimated monochromatic light, usually the mercury green line (5460.73Å), is used. The polarizer, a Glan-Thompson or a Nicol prism mounted in a graduated circle, serves to polarize the light emitted by the source. The compensator, also mounted in a graduated circle, is a birefringent plane usually of quarter-wave thickness; it is used to convert the linearly polarized light into elliptically polarized light. The light incident upon the sample has an azimuthal angle and an ellipticity predictable from the settings of the polarizer and compensator. In general, the light will have its ellipticity and azimuth changed by reflection from the sample.

The “aperature” is an adjustable opening allowing a variation in the area of surface examined and the amount of light reaching the phototube. The analyzer, a second prism in a graduated circle, can be rotated until a minimum intensity is achieved, as indicated by the photometer. If some ellipticity exists in the light reaching the analyzer, extinction of the light cannot be obtained by rotation of only the analyzer. The polarizer and analyzer are then adjusted alternately to remove this ellipticity and obtain extinction. When this occurs, the light reflected from the sample is plane polarized and can be thereby extinguished by the analyzer. The phototube and photometer permit determination of the null point with a high degree of sensitivity. For more accurate null point determinations graphical plots of the intensity of the transmitted beam versus angle of polarizer and analyzer may be used [11].

### 2.2. Alinement

Alinement of the ellipsometer is not too critical for the measurement of thickness and refractive index of thin films. Here the important quantity is the change in readings of the polarizer and analyzer as compared to the values for the bare substrate. However, for obtaining accurate values of the optical constants of surfaces, alinement *is* critical. Moreover, during alinement certain confusing phenomena may occur, and it is worthwhile here to develop the theory of the alinement process.

The procedure for alining the ellipsometer is typical of that generally used in optical spectrometers except for the adjustment of the analyzer (*A*) and polarizer (*P*) scales. When alined, the scales for the polarizer and analyzer read zero when the planes of transmission of the prisms are parallel to the plane of incidence. This is accomplished by first adjusting the polarizer and analyzer prisms in their scales so that these scales differ in setting by 90° when the prisms are crossed. These scales, imagined as a unit, must then be rotated so that when the *P* and *A* scale read 0 and 90° respectively, the planes of transmission of the two prisms are respectively in the plane of incidence and normal to the plane of incidence. That is, the coordinate system defined by the 0 and 90° settings of both *P* and *A* must be made to correspond to the coordinate system defined by the plane of incidence and the plane of the surface.

In principle, the adjustment is relatively simple. With the compensator removed and the polarizer and analyzer arms in the ‘straight-through’ position, the polarizer and analyzer prisms are adjusted in their respective scales until extinction is achieved with the scale readings differing by 90°. The arms are then set for reflection from a metal surface. A minimum in the photometer reading is sought at which the scale readings differ by 90°. The planes of transmission of the two prisms should then be in the plane of incidence and normal to it. The prisms may now be rotated in their holders until the scale readings are 0 ± 180°, and 90 ± 180°, and the alinement is complete.

In practice, however, certain subtle effects occur. After having first crossed the prisms when reflecting from the surface, a minimum may be achieved either by fixing *P* and adjusting *A*, or vice versa. If the light issuing from the polarizer is accurately plane polarized, then the minimum achieved by either of these two means, and with the *P* and *A* scales crossed, occurs at a single value of *P* and *A* independent of the procedure followed. However, if the light issuing from the polarizer has some slight ellipticity due to some imperfection in the polarizer, then there are *two* positions at which the prisms are crossed and minimum photometer readings are obtained, depending upon whether the minimum is achieved by fixing *P* and adjusting *A* or vice versa. Moreover, the deepest minimum or most complete extinction of the reflected light now occurs at a third point, at which the scales are not crossed. Fortunately, with certain assumptions, even in this case alinement may be achieved quite accurately. We shall first give a theoretical explanation of the phenomena observed, and then give a procedure for alining the ellipsometer.

We shall assume that the polarizer produces light of a constant ellipticity χ whose azimuth changes as the polarizer is rotated. The analyzer is taken to be perfect. It is assumed that the *P* and *A* prisms have been adjusted without reflection so that when minimum transmission is achieved the scale readings differ by 90°. The compensator is removed from the system. The process will be depicted on the Poincaré sphere. We consider only ellipticity and azimuth much smaller than unity. In this case the Stokes parameters given by eq (6) are approximated by

$$\begin{array}{l} s_{1}=1\\ s_{2}=2\alpha \cos\delta =2\varphi \\ s_{3}=2\alpha \sin\delta =2\chi . \end{array}$$ (15)

Hence the state of polarization may now be represented by the plane polar coordinates 2*α* and *δ*, and this corresponds to representing as a plane surface the portion of the sphere on the equator around the azimuth representing the plane of incidence, i.e., around *I_p_*.

Under the above assumptions, the states of polarization of the light issuing from the polarizer all lie on the line

2αsinδ=2χ (16)

$$P=\frac{\chi }{\tan\delta }.$$ (17)

Any point $P_{1}^{0}$ with the polar coordinates 2*α, δ*, on this line is rotated and translated by the process of reflection to the point *P*′ with coordinates *2α', δ*′, with the relations

$$\begin{array}{l} 2\alpha^{\prime }=\frac{2\alpha }{\tan\psi }\\ \delta^{\prime }=\delta +\Delta . \end{array}$$ (18)

**Figure 6:**
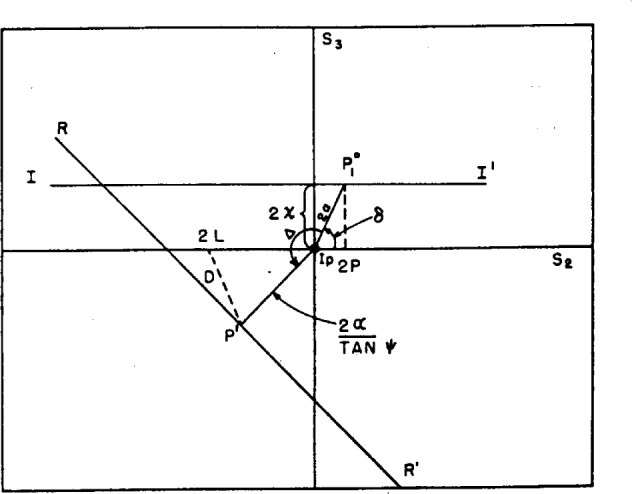
The Poincaré representation of metallic reflection for light of very small azimuth and ettipticity caused by an imperfect polarizer. The point along *I*–*I*′ such as *P*_1_^0^ is translated to a point on *R–R*′ such as *P*′ by the process of reflection. This is passed through as analyzer with plane of transmission at an azimuth *L*+π/2. The amplitude of the light issuing from the analyzer is proportional to *D*.

This process is illustrated in figure 6, where *I–I*′ represents the locus of points representing the incident light and *R–R*′ the reflected light.

The state of polarization of the light after reflection, represented by the point *P*′, may be resolved into components along the plane of transmission of the analyzer and normal to it. The latter direction is represented in the figure by the point *2L*. Under these conditions of very small azimuth and ellipticity, and by eqs (11) and (2), the amplitude of the light transmitted by the analyzer is proportional to the distance *D* between *P*′ and *2L*.

By the triangle *P*′, 2*L, I_p_*, the intensity of the transmitted light is proportional to

$$D^{2}=4L^{2}+\frac{4\chi^{2}}{\tan^{2}\psi \sin^{2}\delta }-\frac{8L\chi }{\tan\psi }\left(\frac{\cos\Delta }{\tan\delta }-\sin\Delta \right)$$ (19)

where 2*L* is to be considered an algebraic quantity, negative in figure 6.

The process of taking readings may be accomplished in two ways. First, the azimuth of *P* may be set and *A* adjusted for minimum transmission. This is equivalent to fixing *δ* and adjusting *L*, i.e.,

$$\frac{\partial \left(D^{2}\right)}{\partial L}=0,$$

whence

Ltanψ=χ(cosΔcosδ−sinΔ)

or

$$\left(A+\frac{\pi }{2}\right)\tan\psi =P\cos\Delta -\chi \sin\Delta$$ (20)

which is the equation of the locus of points obtained in this manner.

**Figure 7:**
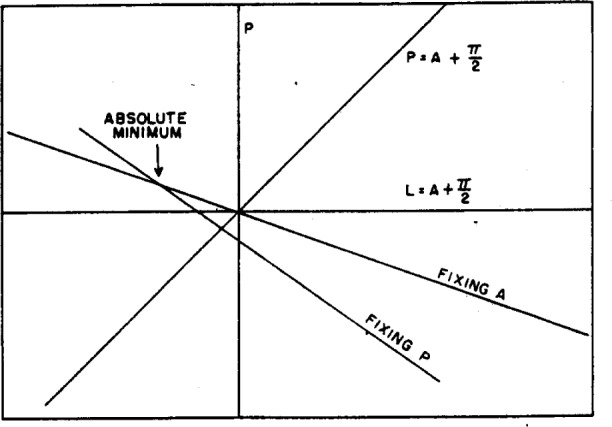
*The locus of points* P *and* A *representing extinction of reflected light for an imperfect polarizer and perfect analyzer*. The upper line is achieved by fixing *A* and adjusting *P* for each setting, and the lower by fixing *P* and adjusting *A* for each setting. Note that the upper line passes through zero azimuth.

Equation (20) is plotted in figure 7. The polarizer and analyzer will be crossed for only one point on this curve. For this point, $P=A+\frac{\pi }{2}$, and

$$P=\frac{\chi \sin\Delta }{\cos\Delta -\tan\psi }$$ (21)

and this crossed position is in general not at zero azimuth $\left(\delta =\frac{\pi }{2}\right)$, but is determined by the constants Δ and *ψ* of the surface.

However, the scales may also be adjusted by first setting *A* and moving *P*. In this case, the condition for a minimum in transmitted light is

$$\frac{\partial \left(D^{2}\right)}{\partial \delta }=0,\;L\tan\;\psi \tan\;\delta \;\cos\;\Delta =\chi$$

and

$$\left(A+\frac{\pi }{2}\right)\tan\psi \cos\Delta =P$$ (22)

which is different from eq (20). The point for which *P* and *A* are crossed is given by

P(1−tanψcosΔ)=0. (23)

Since the quantity in parentheses is not in general zero,

P=0. (24)

Thus it seems clear that the crossed position of the prisms achieved by setting *A* and adjusting *P* for a minimum will always be the true azimuth under these circumstances, i.e., an imperfect polarizer, but perfect analyzer. This is represented in figure 7, where we show both the line achieved by fixing *P* and adjusting *A* and the line achieved by fixing *A* and adjusting *P*. These curves were calculated from eqs (4) and (6) for Δ=135 deg and tan *ψ*=½, and *P* and *A* are in the units of χ. It will be noticed that the curve obtained by first fixing *A* passes through the zero azimuth.

**Figure 8:**
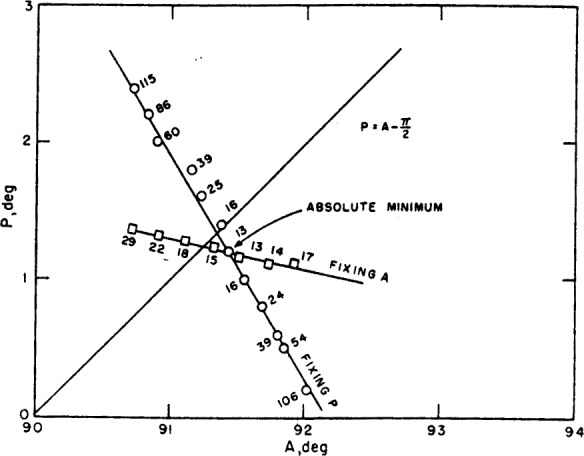
*The experimental counterpart of* figure 7. The *P* and *A* coordinates of the intersection of the line *P=A−π/2* and the “fixing *A*” line represent the azimuth of the plane of incidence and the plane of the surface, respectively.

For-comparison, a curve obtained experimentally is shown in figure 8, where the numbers indicate meter readings and hence are proportional to the transmission. Since the axes of figure 8 are inverted with respect to figure 7, the ellipticity of the polarizer must be negative. However, the similarity is striking. The value of χ computed from this figure and eq (21) is −0.18 deg.

It will be clear from a consideration of eqs (20), (22), and (12), that it is best to use a polarization azimuth near the plane of incidence rather than near the plane of the surface, since tan *ψ* is generally less than unity, and this has the effect of magnifying the angular difference between *P* and *A±π/*2 by an amount 1/tan *ψ*. If the polarizer were set near the plane of the surface the angular spread between *P* and *A+π/*2 would be decreased by an amount tan *ψ*, thus decreasing experimental precision.

The condition for the deepest minimum or best extinction is

$$\frac{\partial \left(D^{2}\right)}{\partial L}=\frac{\partial \left(D^{2}\right)}{\partial \delta }=0$$

and is thus given by the simultaneous solution of eqs (20) and (22). This gives

$$A+\frac{\pi }{2}=\frac{-\chi }{\tan\psi \sin\Delta }$$ (25)

$$P=\frac{-\chi }{\tan\Delta }$$ (26)

By eqs (17) and (26)

tanΔ=−tanδ

or

δ+Δ=π (27)

so that the deepest minimum occurs when the reflected light is linearly polarized, as would be expected. This minimum is shown in figures 7 and 8 by the intersection of the two lines. The absolute minimum determined by alternate adjustment of *P* and *A* is the same as this point of intersection within experimental error. It will be noticed that this point is *not* on the line $P=A+\frac{\pi }{2}$. However, if χ=0, then the deepest minimum occurs at zero azimuth for both *P* and *L*, and thus either method of alinement may be used if the light is perfectly plane polarized. Thus, there appears to be only one unambiguous way of alining the ellipsometer when the light from the polarizer has ellipticity, namely, to seek that point at which both $P=A\pm \frac{\pi }{2}$ and minimum transmission is obtained when *A* is set and *P* adjusted.

### 2.3. Alinement Procedure

A step-by-step procedure for alinement of the ellipsometer is as follows:

First, remove the quarter-wave plate. Lower the arms to the “straight through” position. Set the *P* scale to read zero or any convenient value. Adjust *A* until minimum transmission is achieved. The *A* scale will now in general read (*P*±90)+ϵ. Rotate the *A* prism in its holder until ϵ is zero. The *P* and *A* scales are now crossed when the prisms are crossed. The scales should track within ±0.02 deg throughout one revolution.

With a metal surface set for reflection, raise the arms to an angle near the principal angle. Set *A* so that the plane of transmission of the analyzer is approximately in the plane of the surface. Adjust *P* until minimum transmission is achieved, and note the readings of the *P* and *A* scales. Move *A* by 0.1 deg and again adjust *P* and note the values. If the meter reading is higher than for the previous set of values, adjust *A* by 0.1 deg in the other direction. If it is lower, continue in the same direction. Take a series of readings in this manner, changing *A* by 0.1 deg and adjusting *P* for minimum transmission at each setting of *A*, making sure that the settings encompass the lowest meter reading.

Plot the values of *P* and *A* and determine the readings of the *P* and *A* scales at which *P=A±*90*°*. At this point the plane of transmission of the *P* prism is in the plane of incidence and the plane of transmission of the *A* prism is in the plane of the surface. However, the scale readings will in general not be some multiple of 90°. Let the values of *P* and *A* at this point be, for example, 0°+ϵ′ and 90°+ϵ′ respectively. The quantity ϵ*′* is now the amount by which both scales are displaced from the true zero azimuth. Set *A* at the value obtained from the graph. Adjust the *P* prism in its holder until ϵ′ is zero by turning the prism a small amount in its holder and again adjusting the *P* scale for minimum transmission. This will require a series of adjustments. (It is imperative that the *A* scale and prism not be moved at this stage and that the adjustment be done in this way. If, for example, the *P* scale were held and *A* adjusted, the ellipsometer would be alined to the value of $P=A\pm \frac{\pi }{2}$ obtained by fixing *P* and adjusting *A*, which has been demonstrated to be incorrect.) Having adjusted ϵ′ to be zero, remove the reflecting surface and lower the arms to the “straight through” position. Set the *P* scale at zero and adjust the *A* scale for minimum. The *A* scale will now read 90+ϵ′. Tum the *A* prism in its holder until ϵ′ is zero. The ellipsometer *P* and *A* scales are now alined.

This procedure eliminates alinement errors due to ellipticity produced by the polarizer. The effect of ellipticity m the analyzer on alinement has not been determined but is expected to be small since the analyzer is used for extinction of the light. Thus, any ellipticity by the analyzer after extinction of the light will have no effect on the photometer reading.

### 2.4. Determination of Δ and *ψ* From Ellipsometer Scale Readings

By eq (7) the reflection of light from a surface is characterized by the complex reflection coefficient tan *ψe^j^*^Δ^, and hence by the two quantities *ψ* and Δ. It will later be shown how the refractive index of a surface and the thickness and index of films on a surface are calculated from values of Δ and *ψ*. However, the determination of Δ and *ψ* from *P* and *A* readings merits some discussion, for this is not always a perfectly direct matter.

For any given surface there is a multiplicity of polarizer, analyzer, and compensator scale settings that produce extinction by the analyzer of the reflected light from the surface, and it becomes somewhat of a problem to determine the values of Δ and *ψ* from these various readings. In order to explain how these numerous readings arise and how Δ and *ψ* may be computed from them it is well to keep two facts in mind: (a) all azimuthal angles are measured positive counter-clockwise from the plane of incidence when looking into the light beam, and (b) the compensator, which may be set at any azimuth, is generally set so that its fast axis is in an azimuth of ± π/4. The present discussion is for an instrument such as that shown in figure 5 with the compensator before reflection. If the compensator is placed after reflection, suitable corrections will have to be made.

The various readings fall into four sets called zones, two with the fast axis of the compensator set at π/4, numbered 2 and 4, and two with it set at − π/4, numbered 1 and 3. In each zone there is one independent set of polarizer and analyzer readings, making four independent sets of *P* and *A* readings in all. However, since both analyzer and polarizer may be rotated by π without affecting the results, there are 16 polarizer and analyzer settings falling into four independent zones. Since the compensator may also be rotated by π without affecting the results, there are 32 possible sets of readings on the ellipsometer.

Rather than calculating Δ and *ψ* directly from the *P* and *A* values, it is useful to calculate three other quantities, *p, a_p_*, and *a_s_* from the *P* and *A* values, *p* being related to the *P* readings, *a_p_*, related to the *A* readings in zones 1 and 4, and *a_s_* related to the *A* readings in zones 2 and 3. For a perfect quarter-wave plate these are related to Δ and *ψ* by the eq (2)

$$\Delta =\frac{1}{2}\pi +2p$$ (28)

$$\psi =a_{p}=a_{s}.$$ (29)

If the compensator is not a perfect quarter-wave plate, the relationships are

−tanΔ=sinδcot2p (30)

$$\tan^{2}\psi =\tan a_{p}\tan a_{s}$$ (31)

where *δ* is the relative retardation of the compensator. If the retardation *δ* is near π/2, then, to first order, eqs (28) and (30) are the same. However, it is now necessary to distinguish between *a_p_* and *a_s_*. The relationship of *p* and *a_p_* and *a_s_* to the *P* and *A* readings observed on the ellipsometer is best derived by a consideration of the process as represented on the Poincaré sphere, and since this has been adequately treated by Winterbottom [2], we shall merely list the results. The meanings of *p, a_p_*, and *a_s_* in the four zones are as follows:

*Zone* 1. The fast axis of compensator is at −π/4. The polarizer plane of transmission makes an angle of *+p* with the plane of incidence. The analyzer plane of transmission makes an angle of +*a_p_* with the plane of incidence.
*Zone* 2. The fast axis of the compensator set at +π/4. The polarizer plane of transmission makes an angle of *−p* with the surface (an angle of π/2*−p* with the plane of incidence). The analyzer plane of transmission makes an angle of +*a_s_* with the plane of incidence.
*Zone* 3. The fast axis of compensator is at —π/4. The polarizer plane of transmission makes an angle of *+p* with the surface (an angle of *p+*π/2 with the plane of incidence). The analyzer plane of transmission makes an angle of *−a*, with the plane of incidence.
*Zone* 4. The fast axis of compensator is at *+*π/4. The polarizer plane of transmission makes an angle of *−p* with the plane of incidence. The analyzer plane of transmission makes an angle of *−a_p_* with the plane of incidence.

The meanings of *p, a_p_*, and *a_s_* are now clear. The first is the angle between the polarizer plane of transmission and either the plane of incidence or the plane of the surface, while *a_p_* and *a_s_* represent the angle between the analyzer plane of transmission and the plane of incidence, being labeled either *a_p_* or *a_s_* according to whether *p* is the angle between the polarizer plane of transmission and the plane of incidence or the plane of the surface, respectively. With these relationships, it is a simple matter to compute the relationship of *P* and *A* to *p* and *a_p_* or *a_s_*, and hence to Δ and *ψ*, by means of eqs (28) and (29), or (30) and (31). These relationships are summarized in table 1.

For the purposes of this table, it has been assumed that the *P, A*, and compensator scales have been adjusted to read angles with respect to the plane of incidence as previously defined. If the instrument is not arranged in this manner, it is necessary to adjust the readings before using table 1.

The table gives the 16 possible readings for the two settings of the compensator. When working with a completely unknown surface it is still not a simple matter to identify the *P* and *A* readings with one of the entries on this table and hence to compute *p, a_p_*, or *a_s_*. As a first step in this identification it is worthwhile to consider the ranges for Δ and *ψ* and hence for *p, a_p_*, or *a_s_*. For all surfaces, including multiple reflections,

$$0<\psi <\frac{\pi }{2}.$$ (32)

In fact, in the large majority of cases

$$0<\psi \leq \frac{\pi }{4}$$ (33)

and from eq (29) the range of *a_p_* and *a_s_* is the same. For the special case of reflection from a dielectric surface at an angle of incidence smaller than the Brewster angle, Δ= *−π*. For all other cases

0≤Δ≤2π (34)

which gives as a range for *p*,

$$-\frac{\pi }{4}\leq p\leq \frac{3\pi }{4}.$$ (35)

When working with a completely unknown surface, and particularly if multiple reflections are used, it is best to take a complete set of 16 readings for the two settings of the compensator. Then, with the relations (33) and (35), and the further condition that the values of *p, a_p_*, and *a_s_* calculated from these readings must be approximately the same, it is a simple matter to identify each of the scale readings with one of the possibilities outlined in the table. To continue to do this when the surface is known would be redundant and one reading in each zone is sufficient. We have found that the average of one reading in each of two zones with the same compensator setting gives approximately the same result as the average of one reading in each of all four zones.

### 2.5. Differences Among the Zones

In practice, the values of *p, a_p_*, and *a_s_* found from the *P* and *A* readings in the various zones are not identical. A typical set of values is shown in table 2. Differences of as much as 3 deg occur in the values of *p* and somewhat less in the values of *a_p_* and *a_s_*. On the other hand, it will be noticed that the average of the observed values of *p* in zones 1 and 3 is the same as the average of the value obtained in zones 2 and 4. This strongly implies the following equations

$$\begin{array}{cc} p_{1}=p+\delta & p_{4}=p+\delta^{\prime }\\ p_{3}=p-\delta & p_{2}=p-\delta^{\prime } \end{array}$$ (36)

where δ and *δ′* are error terms. These error terms are not constant from run to run, but are usually 1 to 1.5 deg as in table 2. (In this particular set, *δ* and *δ′* are approximately the same. This is not true in general). They are not caused by misalinement or any other readily detectable cause, and their source remains obscure. However, among very many experiments, not a single one has been found where the averages from zones 1 and 3 did not check the average from zones 2 and 4 within experimental error.

The situation for *a_p_* and *a_s_* is not quite so clear-cut. If the compensator is not a perfect quarter-wave plate, then differences in *a_p_* and *a_s_* are to be expected. In fact, it may be shown that for a compensator with retardation *θ* near π/2,

$$a_{p}-a_{s}=(\theta -\pi /2)\cos\;2p\;\sin\;2\psi$$ (37)

where, for the purposes of this calculation, we have used *ψ=a*. Again, an inspection of the table indicates that the average of *a_p_* and *a_s_* from zones 1 and 3 is equal to the average of *a_p_* and *a_s_* from zones 2 and 4 which again strongly implies

$$\begin{array}{cc} a_{1}=a_{p}+\delta & a_{4}=a_{p}+\delta^{\prime }\\ a_{3}=a_{s}-\delta & a_{2}=a_{s}-\delta^{\prime } \end{array}$$ (38)

where the *a’s* with the numerical subscript are observed values in the various zones. Many experiments indicate that these equations seem to be followed quite faithfully. The values of *δ* and *δ*′ in eqs (38) are, in general, different from those in eqs (36).

It will be observed that the equations are not independent, and they cannot be solved for *a_p_* and *a_s_* directly from the observed values of *a*. Fortunately, for a small value of *a_p_−a_s_*, and for *ψ* not too close to zero it may be shown that

$${\left(\tan a_{p}\tan a_{s}\right)}^{1/2}=\tan\frac{\left(a_{p}+a_{s}\right)}{2}$$ (39)

to first order in *a_p_−a_s_*. Thus rather than using eq (31) for computing *ψ*, the above equation is used. This requires a compensator not too different from quarter-wave, and independent measurements indicate that the compensator used in this work has a retardation which differs from *π*/2 by about 1 deg.

Because the averages from zones 1 and 3 check so closely the averages from zones 2 and 4 for both *p* and *a*, measurements are usually taken in zones 1 and 3 only and averaged.

### 2.6. Surfaces

The substrates most easy to work with are those with high reflectance, such as metals. Both smoothness and flatness are factors that must be considered in the selection of a substrate. Irregularities that are small compared to the dimensions of the light beam, which is commonly of the order of 1 mm^2^, are averaged and do not affect the results [4]. Long range regularity (flatness) is desirable if more than one location on a specimen is to be studied. An indication of the regularity is obtained by determination of *p* and *a* at several points on the specimen. Measurements on 1-in. long steel gage blocks of 0.09 *μ* in. tolerance varied only about 0.1° in the polarizer readings from top to bottom, and less in analyzer readings. Acceptable slides prepared by shearing small rectangles from a large ferrotype plate varied about 0.2° for similar lengths and these make convenient and readily available surfaces for study.

Various techniques have been used for the preparation and cleaning of the substrate surface. The chrome ferrotype plate slides used in these investigations were washed in warm distilled organic solvents to remove organic contamination. Since the resulting surface was hydrophobic, the slides were further cleaned with warm chromic acid cleaning solution followed by several washings in warm distilled water. This treatment should not appreciably affect the character of the chromium-chromium oxide surface, but it did remove organic contamination, the surface now being hydrophilic. However, in less than 1 hr the surface became sufficiently contaminated to become hydrophobic, even when enclosed in a covered container. These precleaned slides were passed through a flame immediately before use to remove this contamination; this flaming of the slides restored the hydrophilic character to the surface. For studies under liquids, the slides were placed under the liquid while still warm from the flame. The cleaning of surfaces by flaming has been described by Patrick [12] and Bartell and Betts [13].

### 2.7. Cells

**Figure 9:**
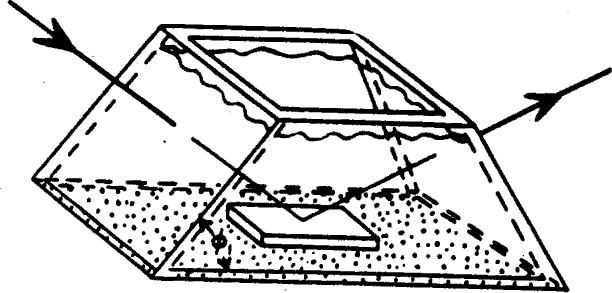
A cell used for studies of surfaces under liquids.

Cells have been used for ellipsometer measurements in vacuum or gaseous environments [2, 14, 15] and under liquids [2]. The cell shown in figure 9 has been used in our studies for measurements under liquids. The specimen is situated on the base of the cell which is filled with liquid of known refractive index. The light beam enters and leaves the cell through optically flat windows. These windows are inclined at the angle of incidence *ϕ* with respect to the base of the cell so that the light passes through them at normal incidence. Therefore, reflection of light at the surface of the cell windows will be independent of its direction of polarization and the polarization of the light will not be changed. There should be no stresses in the glass sufficient to cause detectable birefringence; moreover, the inner and outer sides of each window should be parallel. They may be sealed to the cell body either by fusion or by epoxy resin; the epoxy seal has been used more successfully for our cells. The differences in polarizer and analyzer readings in air for a metallic reflecting surface outside and inside the cell were no larger than 0.1° for our cells.

Difficulties with photometer fluctuations have occurred as a result of convection currents caused by evaporation of liquid. A small area of liquid in contact with the air, a tightly fitting coyer, and solutions filtered through fritted glass disks minimize this effect.

**Figure 10:**
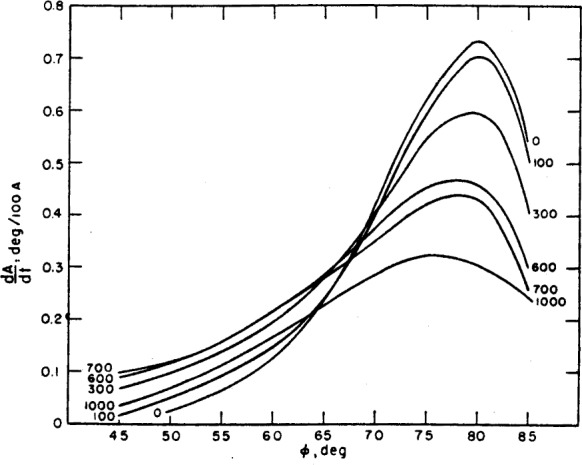
*The sensitivity*, dA/dt, *of* A *for measuring thickness* t, *plotted as a function of angle of incidence*. The parameters are those for a typical organic film on chrome; *n*_1_=1.4268, *n*_2_=1.4700; *n*_3_=3.14800-4.14279*j*.

**Figure 11:**
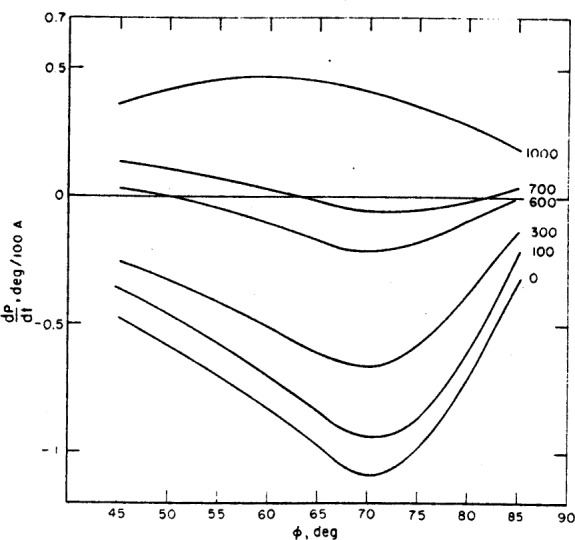
*The sensitivity*, dP/dt *of* P *for measuring thickness* t, *for the same conditions as* figure 10. Note the very low sensitivity at a film thickness of 700 Å.

The angle of incidence should generally be chosen to give the maximum sensitivity for the measurement of film thickness. For this purpose, sensitivity may be defined as the change of *P* reading or *A* reading with film thickness, *t*, i.e., *dP/dt* and *dA/dt*. One is then faced with the problem of. selecting an angle of incidence such that these two quantities are a maximum. No general rules can be laid out for this selection, and the choice of angle of incidence will depend upon the particular substrate, film and surrounding medium. However, for the common case of an organic film on a chromium surface in an organic medium, the sensitivity for both *P* and *A* has been calculated for various angles of incidence and film thicknesses up to 1000 Å. The results are shown in figures 10 and 11.

The maximum sensitivity for *A* occurs at angles of incidence between 75 and 80 deg, and decreases with film thickness. For *P*, the maximum sensitivity (the absolute value of *dP/dt*) occurs at an angle of incidence of 70°, and decreases with increasing film thickness up to a thickness of about 700 Å. At this thickness, the sensitivity in *P* is very small, making the thickness determination entirely dependent on *A*, which is relatively insensitive in this region. Thus, under these conditions, the accurate determination of film thickness is difficult for films 600 to 800 Å thick. For films thicker than 800 Å, the sensitivity in *P* again becomes usable, but the maximum occurs at angles less than 70 deg.

For any film thickness, therefore, the best angle of incidence is always a compromise between the most sensitive angle for *P* and that for *A*. The selection will, in part, depend on the relative importance of *P* or *A* to the determination of the thickness.

These sensitivities apply only to the given specific conditions but the sensitivities for other conditions with a nonabsorbing film are expected to have similar behavior although specific values will be different.

### 2.8. Methods of Computation

**Figure 12:**
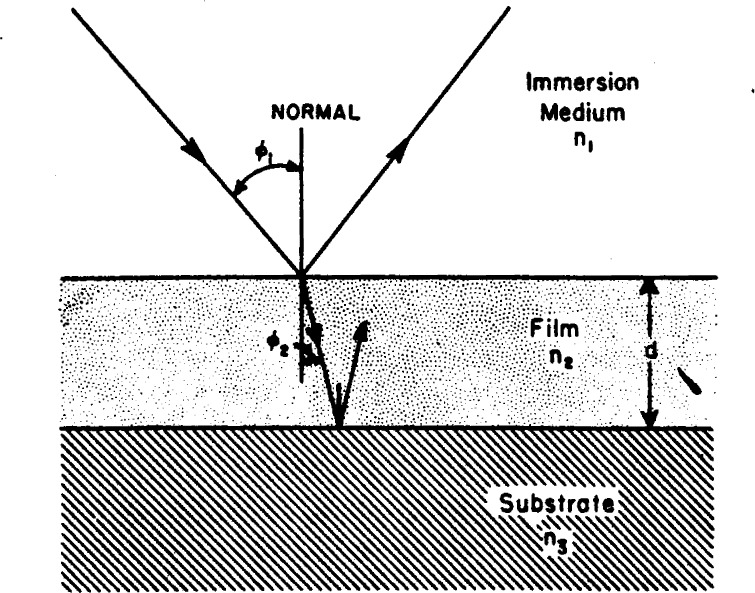
*Reflection from a film-covered surface*. The substrate is a reflecting metal surface.

A typical system for study by the ellipsometer consists of a film of index *n*_2_ and thickness *d* on a reflecting substrate of index *n*_3_ immersed in a medium of index *n*_1_ as shown in figure 12. Let all media be isotropic and *n*_1_ represent a real index of refraction, while *n*_2_ and *n*_3_ may be complex.

Consider light incident at the Doundary between the immersion medium and film. The cosine of the refraction angle is

$$\cos\varphi_{2}={\left[1-{\left(\frac{n_{1}}{n_{2}}\sin\varphi_{1}\right)}^{2}\right]}^{1/2}.$$ (40)

The parallel and normal reflection coefficients for light incident at this boundary are:

$$r_{12}^{p}=\frac{n_{2}\cos\varphi_{1}-n_{1}\cos\varphi_{2}}{n_{2}\cos\varphi_{1}+n_{1}\cos\varphi_{2}}$$ (41)

and

$$r_{12}^{s}=\frac{n_{1}\cos\varphi_{1}-n_{2}\cos\varphi_{2}}{n_{1}\cos\varphi_{1}+n_{2}\cos\varphi_{2}}$$ (42)

respectively. The reflection coefficients, $r_{23}^{p}$ and $r_{23}^{s}$ at the boundary between the film and substrate are given by similar expressions.

The total reflection coefficients, *R^p^* and *R^s^* that include the contributions of reflections from lower boundaries are given [2] by:

$$R^{p}=\frac{r_{12}^{p}+r_{23}^{p}\exp D}{1+r_{12}^{p}r_{23}^{p}\exp D}$$ (43)

and

$$R^{s}=\frac{r_{12}^{s}+r_{23}^{s}\exp D}{1+r_{12}^{s}r_{23}^{s}\exp D}$$ (44)

where cos *φ*_3_ values needed for these reflection coefficients are given by an expression similar to eq (40) and *D* represents the quantity

$$D=-4\pi jn_{2}\cos\varphi_{2}d_{2}/\lambda$$ (45)

where λ is the wavelength of the light used, in vacuum, and $j=\sqrt{-1}$. The ratio of the parallel and normal total reflection coefficients is defined as *ρ*:

$$\rho =R^{p}/R^{s}.$$ (46)

This may be expressed in terms of the relative attenuation and phase shift of the parallel component with respect to the perpendicular component that occurs, represented by the azimuthal angle Δ, and relative phase shift *ψ* by:

ρ=tanψexp(jΔ) (47)

as in eq (8). Thus *ρ* is determined from ellipsometer readings.

The value of the complex index of a reflecting surface can be calculated from the equation

$$n_{3}=n_{1}\tan\varphi_{1}{\left[1-\frac{4\rho \sin^{2}\varphi_{1}}{{\left(\rho +1\right)}^{2}}\right]}^{1/2}$$ (48)

where *φ*_1_ is the angle of incidence and *ρ* is determined from ellipsometry measurements on the base substrate.

**Figure 13:**
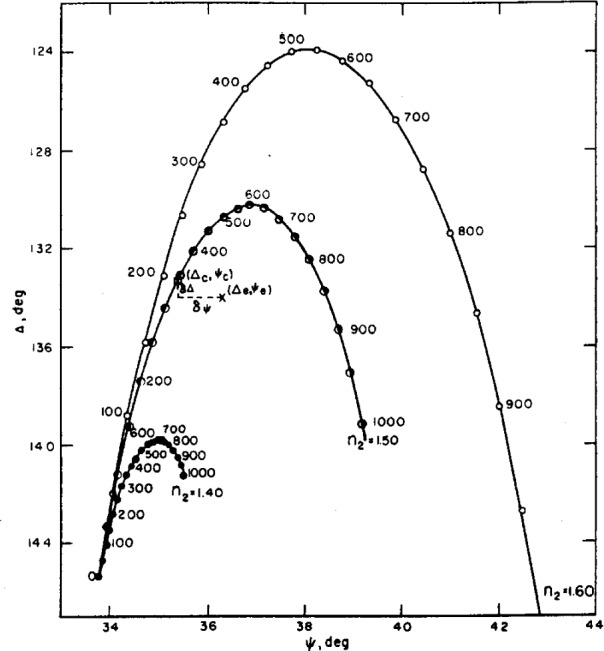
*A plot of* Δ *against ψ for films of refractive index as shown, in a medium with a refractive index of 1.359*. The numbers along the curves are film thicknesses In Angstrom units. From such a figure and an experimental point *ψ_e_*, Δ*_e_*, both the retractive Index and thickness of the film may be obtained (see text).

Several methods of determining the thicknesses of films on reflecting substrates from ellipsometry measurements are available [2–5]. When the indexes of the substrate and film are known, tables or graphs of Δ and *ψ* may be computed from given values of *d*. This is accomplished by calculating values of *ρ* from eqs (40) to (46) and values of Δ and *ψ* from eq (47). Figure 13 shows curves computed for three different values of film index *n*_2_. The numbers along each curve are thickness values of the film, *d_2_*. Experimental values of Δ and *ψ* are then interpolated in such tables or related graphs to determine unknown thicknesses.

However, it is usually more efficient to solve the equations directly from the thickness of a film. Substituting eqs (43), (44), and (46) in eq (47) and rearranging gives a quadratic of the form:

$$C_{1}{\left(\exp D\right)}^{2}+C_{2}(\exp D)+C_{3}=0$$ (49)

where *C*_1_,*C*_2_, and *C*_3_ are complex functions of the refractive indexes, angles of incidence, Δ *and ψ*. For a given value of the coefficients eq (49) gives two solutions for exp *D* and a value of *d* may be calculated from each. Since the coefficients are complex, the film thicknesses calculated from this equation would also be expected to be complex. However, the correct film thickness, *d*, must be a real number as it represents a real quantity. Therefore, the solution of the quadratic that yields a real film thickness is the correct solution. In practice, various experimental errors will result in both solutions yielding complex values for *d*. The thickness with the smallest imaginary component is selected as the correct solution; the real portion is taken as *d* and the imaginary part, *dj*, is taken as a relative measure of error.

The real portion of *d* is then used to compute Δ and *ψ* by eqs (43) to (47). As the imaginary component of *d* has been dropped, these values will differ from the experimental angles by amounts *δ*Δ and Δ*ψ*, and *d_j_, δ*Δ, and *δψ* are all measures of the experimental error. However, *δ*Δ and *δψ* must be within the limits of experimental error of *ψ* and Δ for the results to be valid. This is a more direct determination of the validity of an experiment than the magnitude of *d_j_*.

If both the thickness and index of refraction of the film, *n*_2_, are not known, the equations cannot be solved for *d* and *n*_2_ in closed form. For this case, a series of refractive indexes are assumed and a thickness is calculated from the experimental measurements. These calculations will result in error terms, described above, of different magnitudes. The range of refractive indexes and related thicknesses within the experimental errors δΔ and *δψ* are then chosen. The magnitude of the possible ranges of refractive indexes and thicknesses will depend upon the magnitude of the error and on several parameters such as *n*_1_, *n*_2_, *n*_3_, etc.

Some of the principles described above are illustrated in figure 13, which shows theoretical Δ and *ψ* values for a range of given film indexes and thicknesses. An experimentally determined point (Δ*_e_*, *ψ_e_*) for a film of unknown *n_2_* falls near one of these curves. For an assumed film index of 1.5, a thickness will be calculated corresponding to the point (Δ*_e_*, *ψ_e_*). The error terms, *δ*Δ and *δψ* are also illustrated. The error terms are usually much smaller than indicated on this figure.

The calculations discussed above can be computed manually, but the equations are complicated and the computation of any appreciable amount of data would be impractical. Several “Fortran” programs have been written in this laboratory for IBM 704 and 7090 computers to enable the computation of data from ellipsometer measurements. The programs are general enough to permit their use in most situations encountered. The following is a brief description of problems that can be solved with these programs.

The refractive index of the substrate, *n*_3_, may be computed directly from experimental readings on the bare substrate. Immersion media of different refractive indexes may be used for this determination.

When *a_p_* and *a_s_*, are known, the correct retardation of the compensator can be calculated. This retardation may then be used in subsequent calculations. Thus, the ellipsometer data may be used even if the compensator is not a quarter-wave plate.

Curves and tables of Δ*ψ*, and reflection coefficients can be computed for a series of given film thicknesses and refractive indexes, *n_2_*, as shown in figure 13.

For experimental values of Δ and *ψ*, a thickness, *d*, and error terms, *δψ* and *δ*Δ, are computed for a given *n*_2_. If *n*_2_ is unknown, a series of *n*_2_ can be assumed and corresponding film thichness and error terms computed. The selection of the correct *n*_2_ and thickness has been discussed and is illustrated in the next section. All the above calculations have been extended for multiple films and for multiple reflections.

## 3. Applications

Inasmuch as surfaces in air will usually have an adsorbed film of water or other contaminants, in order to determine the index of a substrate it is necessary either to measure the surface in a vacuum or in a medium with a refractive index identical to that of the adsorbed film (*n*_1_=*n*_2_).

In the work reported here chrome slides with and without a vacuum deposit of gold were flamed to remove adsorbed gases and immediately immersed in a liquid. Table 3 gives the refractive indexes of slides measured in air and measured under water. The differences in the refractive index of the metals calculated from measurements made in air and under water are probably caused by the presence of an adsorbed film of water on the slides when measured in air. Such a film would not be observed, of course, when the measurements were made under water, assuming the refractive index of the film to be the same as that of water. It is presumed, therefore, that the measurements made under water yielded the correct indexes for these slides.

The measurements described above were also repeated for immersion media of different refractive indexes. The optical constants of the metal calculated under these conditions are given in table 4. The slides measured under liquids were flamed and immediately immersed in the liquid before a film could form on them from the air. These slides are therefore not expected to have any adsorbed film on them, so that their true refractive indexes are, measured. If some film did remain on them, its refractive index would probably be near that of the immersion liquid, causing only a small error in the measured refractive index of the slide. Hence the rerfactive indexes of the slides under the various liquids are all approximately the same, and independent of the liquid and the angle of incidence. The variation is due mainly to variations among the individual slides used for the different measurements.

The refractive index measured in air shows a difference due to neglect of the films that is larger than the variations among the individual slides. This film is expected to be adsorbed water and, perhaps, other gases and is expected to have an index near that of water. By first measuring slides in air and then measuring them under water, it is therefore possible to calculate a thickness of the adsorbed layer in air, assuming the refractive index of the adsorbed film to be the same as that of liquid water. Such results are shown in table 5, where *δ*Δ and δ*ψ* are the differences obtained as described above. The reproducibility of the results is seen to be quite good, although the actual value of the thickness may be somewhat in error due to the assumption of the refractive index value.

The determination of both the thickness and refractive index of a barium fluoride film vacuum evaporated onto a chrome surface is given as an example of the measurements and calculations described earlier. The index, *n*_3_, of the substrate was first determined from the reading taken on the bare chrome surface, then the film was vacuum deposited on the surface and the angles Δ and *ψ* were measured for the film-coverea surface. A series of refractive indexes are assumed for the film, and thickness and the corresponding error terms, *δ*Δ and *δψ*, are calculated for each index by the methods given earlier. The results are given in table 6. The reproducibility of the readings is ±0.1° for Δ and ±0.05° for *ψ* and the maximum experimental error is expected to be about ±0.2° for Δ and ±0.1° for *ψ*. It will be observed from table 6 that the index 1.456 gives zero error terms *δ*Δ and *δψ*, and is, therefore, the best fit with the experimental reading. However, *n*_2_ values from 1.454 to 1.458 also give error terms within the limits of experimental error, and are the range of possible indexes of the film. The corresponding thicknesses are 654 and 665 Å.

**Figure 14:**
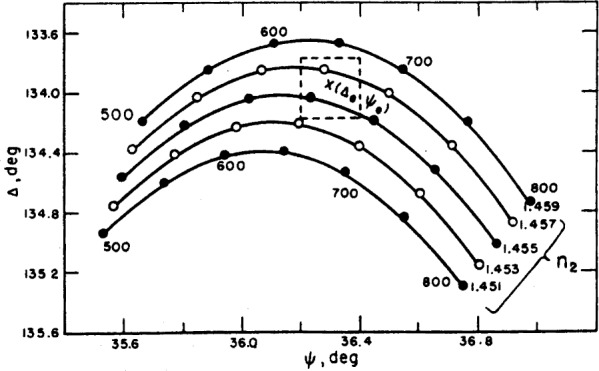
*An enlargement of* figure 13, showing how error in Δ*_e_ and ψ_e_*, *leads to an uncertainty in the calculated refractive index and thickness of a film*.

The actual range of possible thicknesses is greater than this range as is illustrated in figure 14. This figure is an enlargement of the center portion of figure 13. Curve3 for additional refractive indexes have been added. The dashed lines represent the limits of experimental error for the point (Δ*_e_*, *ψ_e_*). The true values of *n*_2_ and *d* must fall within these limits. A range of possible thicknesses for each refractive index rather than only a single selected value of thickness for each possible refractive index is obtained. The range of possible thicknesses is thus approximately 630 to 680 Å.

These methods Ho not determine the index of the film as accurately when the film is very thin. This can be observed in figure 13 by the close proximity of the curves for small thicknesses. Since the experimental error is independent of film thickness, the range of refractive indexes and thicknesses that would be included in the rectangle of experimental error described above is increased. When the refractive index is known, the thickness can be accurately determined even for very thin films, except that as the film index approaches that of the medium, the sensitivity in determining the film thickness decreases. This can be seen in figure 13 by the decreasing sensitivity to thickness of Δ and *ψ* as the refractive index of the film, *n*_2_, approaches the index of the medium, 1.359.

## 4. Summary

The use of the ellipsometer for the measurement of the thickness and index of refraction of very thin films is described and illustrated by examples. The representation of elliptically polarized light by means of the Poincaré sphere is developed in detail since it is necessary for a complete understanding of the changes in the polarization of light in the ellipsometer.

Some unusual effects in alinement have been observed and explained as due to the light from the polarizer having a slight ellipticity. A method of alinement that is not affected by the ellipticity is given.

Chrome ferrotype plates have been found to be a convenient substrate for study of thin films. Means of preparing and cleaning the plates are discussed. Also, cells used to study films under liquids are described.

In order to study a film by ellipsometry, the reflection coefficient of a bare substrate is first measured and the complex refractive index of the substrate computed from the reflection coefficient. A film is then deposited on the substrate and the reflection coefficient of the combination is measured. If the index of the film is known, the thickness of the film may be computed. In one method, the reflection coefficient of the substrate with a film is computed for many thicknesses O_1_ the film. Then the measured reflection coefficient is interpolated in the calculated results to determine the thickness of the film. However, in this paper the required equations are solved to give the thickness of the film directly in terms of the measured reflection coefficients so that interpolation is not required. These measurements and calculations are applied to determining the thickness of an adsorbed film on ferrotype plates. The index of refraction of this film was assumed to be that of water.

Both the index of refraction and thickness of a film may be calculated from the complex reflection coefficient of the film-substrate combination. The procedure is to assume a series of refractive indexes and compute a corresponding thickness for each index. If the refractive index chosen is not the exact index of the film, and if the measurements are not sufficiently precise, the calculated thickness of the film is complex. The imaginary part of the computed complex thickness is taken as a relative measure of error either of the assumed refractive index or of the original measurement. The film refractive index with its corresponding thickness that yields the smallest error terms is taken as the best fit. This calculation is illustrated for a barium fluoride film.

## Figures and Tables

**Table 1:** *Relation of* P *and* A *readings to* p, a*_p_*, *and a_s_*

Zone	Compensator	*P*	*A*
			
1	−*π*/4	*p*	*a_p_*
		*p+π*	*a_p_*
		*p*	*a_p_+π*
		*p+π*	*a_p_+π*
3	−*π*/4	*p*+*π/*2	*π*−*a_s_*
		*p*+3*π/*2	*π*−*a_s_*
		*p*+*π/*2	2*π*−*a_s_*
		*p*+3*π*/2	2*π*−*a_s_*
2	+*π/*4	*π/*2−*p*	*a_s_*
		3*π*/2−*p*	*a_s_*
		*π*/2−*p*	*a_s_*+*π*
		3*π*/2−*p*	*a_s_*+*π*
4	+*π/*4	*π*−*p*	*π*−*a_p_*
		*2π*−*p*	*π*−*a_p_*
		*π*−*p*	2*π*−*a_p_*
		*2π*−*p*	2*π*−*a_p_*

**Table 2:** Values of P and A for a typical chrome slide in air in all four zones

Zone	*P*	*A*	*p*	*a_p_*	*a_s_*
					
1	19.71	31.04	19.71	31.04	
2	167.17	31.73	22.83		31.73
3	113.00	147.75	23.00		32.25
4	160.20	148.52	19.80	31.48	
		
Average of four zones…………			21 34	31.63	
Average of zones 1 & 3…………			21.36	31.65	
Average of zones 2 & 4…………			21.32	31.61	

**Table 3:** *Measured refractive index*, n=N−jK, *of chrome*^a^ *and gold*^a^ *in air and in water*

Immersion medium	Chrome		Gold	
	*N*	*K*	*N*	*K*
				
Air…………	2.954	4.224	0.446	2.134
Water…………	3.231	4.354	.528	2.165
Air…………	3.005	4.274	.427	2.119
Water…………	3.218	4.302	.546	2.100
Air…………	2.950	4.258		
Water…………	3.285	4.426		

a Slides bad been flamed prior to examination.

**Table 4:** *Refractive indexes*, N—jK, *calculated for chrome slides immersed in various liquids immediately after flaming*

*φ*	Immersion medium	*n*	Refractive index^a^	
			*N*	*K*
				
*Degree*				
60	methanol……………	1.329	3.212	4.335
60	water……………	1.337	3.416	4.358
70	water……………	1.337	3.278	4.371
60	acetone……………	1.360	3.275	4.363
70	acetone……………	1.360	3.296	4.398
70	cyclohexane……………	1.426	3.254	4.396
70	toluene……………	1.498	3.344	4.437
60	air^b^……………	1.00	2.899	4.170
70	air^b^……………	1.00	2.918	4.198

a All refractive Indexes are averages of two or more sets of measurements, except for methanol.

b Slides measured in air bad been exposed to air from 1 to 24 hr.

**Table 5:** *Thickness of adsorbed layer*^a^ *on slides in air*

	Slide number	*φ*	Δ	δΔ	*ψ*	*δψ*	*d*
							
		*Degree*	*Degree*	*Degree*	*Degree*	*Degree*	Å
Chrome	76	50	163.44	0.00	39.51	0.04	23
	76	60	152.56	.02	36.33	.11	24
	76	70	132.52	.06	31.85	.13	26
	75	70	132.72	.08	31.57	.16	27
	74	70	131. 78	.04	31.83	.08	24
	79	70	132.04	.06	31.76	.12	23
	76	80	85.04	.06	28.15	.18	26
	
Average……………………………………………………							25
	
Gold	72	70	90.36	0.06	40.53	0.15	2
	73	70	91.96	.00	40.98	.02	3
	74	70	88.38	.12	39.89	.26	5
	77	70	88.36	.06	40.39	.16	4
	
Average……………………………………………………							4

a Calculated as water

*T*=22° C,

*RH*=50 percent.

**Table 6:** Thickness of evaporated barium fluoride film on chrome slide^a^,^b^

Δ	δΔ	*ψ*	*δψ*	*n*_2_	*d*
					
*Degree*	*Degree*	*Degree*	*Degree*		Å
133.96	3.64	36.30	0.60	1.420	753
	2.64		.40	1.430	726
	1.58		.22	1. 440	701
	1.08		.14	1. 445	688
	0.58		.07	1.450	676
	.28		.03	1.453	668
	.18		.02	1.454	665
	.10		.01	1.455	662
	.00		00	1.456	660
	.10		.01	1.457	657
	.20		.02	1.458	654
	.28		.03	1. 459	651
	.38		.04	1.460	647
	.70		.38	1. 475	453

a Immersed in acetone, *n*_1_=1.359, at an angle of incidence of 60°. The refractive index of the slide was *n*_3_=3.316−4.383*j*.

b Film prepared by Frank E. Jones, National Bureau of Standards.

## Footnotes

2 Figures in brackets indicate the literature references at the end of this paper.
